# Simultaneous Association of Variations in the Origin and Diameter of the Left Vertebral Artery in a Patient with a C1 Lateral Mass Tumor

**DOI:** 10.1155/2022/1025019

**Published:** 2022-04-28

**Authors:** Seyed Reza Mousavi, Majid Reza Farrokhi, Shayan Yousufzai, Maryam Naseh, Fatemeh Karimi

**Affiliations:** ^1^Shiraz Neuroscience Research Center, Shiraz University of Medical Sciences, Shiraz, Iran; ^2^Department of Neurosurgery, Shiraz University of Medical Sciences, Shiraz, Iran; ^3^Student Research Committee, Medical School, Shiraz University of Medical Sciences, Shiraz, Iran; ^4^Histomorphometry and Stereology Research Center, Shiraz University of Medical Sciences, Shiraz, Iran; ^5^Anatomy Department, School of Medicine, Shiraz University of Medical Sciences, Shiraz, Iran

## Abstract

The anomalous origin of a hypoplastic Left Vertebral Artery (LVA) from the aortic arch is a rare anatomic variant. This study discusses the case of a patient with a C1 lateral mass tumor that surrounded a dominant Right Vertebral Artery (RVA) according to preoperative computed tomography angiography, with a hypoplastic LVA originating from the aortic arch. Surgery was performed, and the patient recovered uneventfully. To date, no study has reported the simultaneous association of two variations (origin and diameter) in the LVA. A deep understanding of abnormalities in the diameter and origin of LVA is a must for neurosurgeons as well as for thoracic and vascular surgeons to conduct surgical procedures.

## 1. Introduction

The branches of the vertebral artery can vary in origin, diameter, and course [1]. The Left Vertebral Artery (LVA) origin represents one substantial variation that surgeons must be aware of [2]. The prevalence of anomalous vertebral artery origins is higher in the LVA (6%) than in the Right Vertebral Artery (RVA) (3.8%) [1, 3]. The LVA longitude can show different variations, including hypoplasia and termination at the Posterior Inferior Cerebellar Artery (PICA) rather than the basilar artery [2]. Vertebral Artery Hypoplasia (VAH), another common vertebral artery variation, has been considered an anomaly in which the vertebral artery diameter is less than 2 mm [4]. Recent studies have suggested that patients with VAH can show vertebrobasilar system insufficiency symptoms, especially when they have vascular risk factors or when a dominant vertebral artery fails to supply the posterior circulation [5, 6].

The embryological complexity and extensive anatomy of the vertebrobasilar system are responsible for the development of uncommon variations. Congenital vascular defects can strongly affect the outcomes of angiographic and surgical interventions [7]. To date, no studies have reported the presence of a hypoplastic LVA originating from the aortic arch. This case report presents a sporadic case of the simultaneous association of variations in both the origin and diameter of the LVA in a patient with a C1 lateral mass tumor.

## 2. Case Presentation

**Figure 1:**
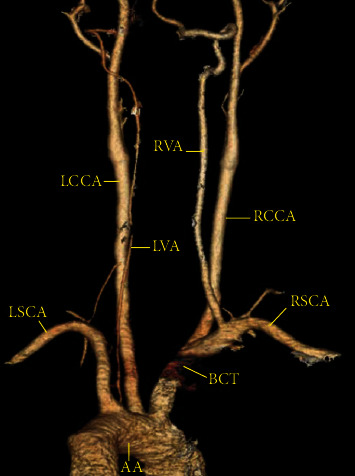
Three-dimensional reconstructed computed tomography angiography shows the carotid, subclavian, and vertebral arteries. A hypoplastic left vertebral artery originates from the aortic arch and is smaller than the right vertebral artery along its entire course. All mentioned vascular structures show normal caliber and course, smooth intima, and no narrowing or obliteration. AA = aortic arch; BCT = brachiocephalic trunk; LCCA = left common carotid artery; LSCA = left subclavian artery; RCCA = right common carotid artery; RSCA = right subclavian artery; LVA = left vertebral artery; RVA = right vertebral artery.

**Figure 2:**
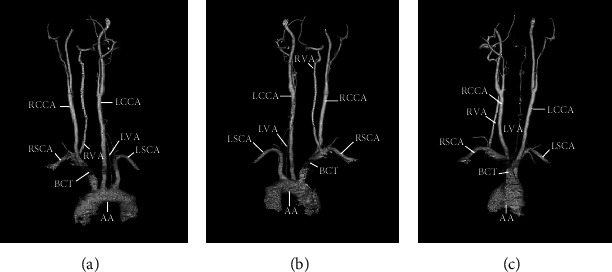
Computed tomographic angiography demonstrates the anterior-posterior (a), posterior-anterior (b), and lateral (c) views. AA = aortic arch; BCT = brachiocephalic trunk; LCCA = left common carotid artery; LSCA = left subclavian artery; RCCA = right common carotid artery; RSCA = right subclavian artery; LVA = left vertebral artery; RVA = right vertebral artery.

A 33-year-old woman complained of intolerable neck pain commencing one month earlier. On physical examination, the patient showed local tenderness in the upper cervical region without any neurological deficits. Through Magnetic Resonance Imaging (MRI), a lateral mass was found on the right side of her first cervical vertebrae. Computed tomography angiography (CTA) was performed to evaluate the cerebral vessels, which revealed variations in the origin and diameter of the LVA [8]. The LVA originated from the aortic arch, between the Left Common Carotid Artery (LCCA) and the Left Subclavian Artery (LSCA) (Figure 1). In addition to the abnormal origin, a hypoplastic LVA was observed (Figure 2). The dominant vertebral artery was the RVA. The RVA diameter was 4.1 mm, and the LVA diameter was 1.5 mm. Therefore, the RVA was significantly larger than the LVA. Additionally, the LVA was shorter than the RVA (16.8 vs. 18.4 cm). The LVA passed through the transverse foramina of the sixth cervical vertebrae and formed the basilar artery by joining the RVA. Other LSCA and RSCA branches were normal, and no specific variations were noted. No evidence of dissection or aneurysmal dilation was detected. In addition, the carotid artery bifurcations showed standard configuration with no filling defects, plaques, or narrowings.

## 3. Discussion

The present study discusses the unprecedented simultaneous occurrence of both hypoplasia and an anomalous origin of the LVA in a patient with chronic neck pain due to a C1 lateral mass tumor. The RVA was dominant and was surrounded by the tumor at the craniovertebral junction.

Vertebral arteries are among the major arteries in the cervical area, typically arising from the first part of the subclavian artery on both sides [1]. Previous studies showed that the LVA was the most common site for variations in the origin of vertebral arteries [9]. The LVA can originate from atypical sites such as the aortic arch, common carotid artery, and internal or external carotid arteries. Furthermore, the LVA can have dual origins from the aortic arch and the subclavian artery [1, 10]. An origin from the aortic arch is a common variation, with a prevalence rate of 2.4-6.9%. However, in most variants, the LVA is situated between the LCCA and the LSCA [11–13].

The variable origin of the LVA carries remarkable importance in surgical and clinical settings. Understanding this issue is necessary for experts involved in the fields of head and neck surgery, cerebral disorders, angiography, arterial dissection, and stent placement in vertebral or carotid arteries [14]. Blumberg et al. reported that an LVA originated from the aortic arch in a patient with an acute intramural hematoma [15]. In another study conducted by Fridah et al., 84 vertebral arteries were evaluated by CTA in a Zambian population, three of which originated from the aortic arch [16]. Yamaki et al. dissected 515 vertebral arteries in Japanese adult corpses, among which 30 LVAs were noted to originate directly from the aortic arch [17]. When the LVA branches from the aortic arch, its opening is exposed to turbulent blood flow, paving the way for iatrogenic injuries [18].

Changes in the site and pattern of branching, agenesis, perforating branches, and hypoplasia are the most reported variations of the vertebral arteries [19, 20]. The prevalence of vertebral artery hypoplasia appears to be 1.9-11.6% [21], though Ogeng et al. monitored 346 vertebral arteries for hypoplasia in Kenya, revealing a prevalence of 28.9% [22]. This latter figure seemed to be higher than those seen in other populations. Researchers have found that vertebral artery hypoplasia is linked to an increase in the chance of posterior circulation ischemia. This finding was further noted in the PICA, where relative hypoperfusion occurs [23]. An interesting study conducted by Harati et al. demonstrated a 52% linkage of vertebral artery hypoplasia with VA-PICA aneurysms. These researchers also emphasized that blood pressure and blood flow were two major factors affecting vascular morphology [24].

To date, several studies have been conducted to evaluate LVA abnormalities. Nonetheless, no study has reported the simultaneous association of abnormal variants in the diameter and origin (aortic arch) of the LVA. The current research is believed to be the first study indicating the simultaneous presence of these two variants in the LVA, which was hypoplastic (with a diameter of 1.5 mm) and originated from the aortic arch.

### 3.1. Embryological Development

In order to appreciate variations in the vertebral arteries, one must inspect the embryological development and branching patterns of the aortic arch. During embryologic development, the intersegmental arteries branching from the dorsal aorta are responsible for supplying the somites and their derivatives [25]. As the human cervical region develops during the embryonic period, longitude anastomosis between the C1 and C7 intersegmental arteries results in the formation of the vertebral arteries. Both the right and left vertebral arteries are derived from the distal portion of the dorsal C7 intersegmental artery [20]. Furthermore, most of the primary connections of the intersegmental arteries with the dorsal aorta disappear. Hence, the remaining primary vessels can develop anatomical variations in the vertebral arteries [26]. In some cases, the anastomosis of the C6 and C7 intervertebral arteries remains incomplete on the left side. Hence, C6 remains free, which causes the LVA to originate from the aortic arch between the LCCA and the LSCA [27]. Patil et al. stated that enhanced embryonic tissue absorbance from the LSCA between the vertebral artery origin and the aortic arch could explain this phenomenon [4]. Nonetheless, further research should be undertaken to determine why some arteries persist and others disappear.

The studies conducted on the embryologic reasons for hypoplasia in the vertebral arteries have underlined four carotid-vertebrobasilar anastomosis types in the early embryonic period, namely, the Proatlantal Intersegmental Artery (PIA), hypoglossal artery, otic artery, and trigeminal artery. It is noteworthy that most of these anastomosis types vanish in one week as the vertebrobasilar arterial system develops. If vertebral arteries do not develop and fail to join the basilar artery, PIA anastomosis continues to persist. However, permanent anastomosis of the PIA is among the important reasons for vertebral artery hypoplasia and agenesis. Some studies have demonstrated the association of PIA anastomosis with posterior circulation infarction, transient ischemic attacks, and vertebrobasilar insufficiency [19]. However, no study has reported the simultaneous association of two variations (origin and diameter) in the LVA. Therefore, further research is strongly recommended in this area.

## 4. Conclusion

It is substantially rare to observe the left vertebral artery being hypoplastic and simultaneously originating from the aortic arch. Overall, a deep understanding of the developmental anomalies in diameter and origin of the vertebral arteries is a must for neurosurgeons as well as for thoracic and vascular surgeons. Our findings can also guide endovascular interventions.

## Data Availability

Patient data were accessed through medical records at Shiraz University of Medical Sciences and are unavailable for release due to patient confidentiality.
